# Diphtheria, tetanus, and pertussis immunity among healthcare professionals and pregnant women in the Moscow region, Russian federation: A preliminary cross-sectional study

**DOI:** 10.3389/fped.2023.1043707

**Published:** 2023-02-21

**Authors:** Artem A. Basov, Yury V. Zhernov, Maria I. Kashutina, Natalia N. Kashkovskaya, Svetlana Yu. Kombarova, Inga I. Enilenis, Lyudmila P. Severova, Inna A. Fadeeva, Sonya O. Vysochanskaya, Elena V. Belova, Ekaterina A. Shashina, Valentina V. Makarova, Denis V. Shcherbakov, Anton Yu. Skopin, Oleg V. Mitrokhin

**Affiliations:** ^1^ Diphtheria and Pertussis Surveillance Laboratory, G.N. Gabrichevsky Research Institute for Epidemiology and Microbiology, Moscow, Russia; ^2^ Department of General Hygiene, F. Erismann Institute of Public Health, I.M. Sechenov First Moscow State Medical University (Sechenov University), Moscow, Russia; ^3^ Department of Chemistry, Lomonosov Moscow State University, Moscow, Russia; ^4^ Center of Life Sciences, Skolkovo Institute of Science and Technology, Moscow, Russia; ^5^ Center for Medical Anthropology, N.N. Miklukho-Maclay Institute of Ethnology and Anthropology of the Russian Academy of Sciences, Moscow, Russia; ^6^ Department of Diagnostics and Treatment of Diseases of the Breast and Reproductive System No 2, Women's Health Clinic – Mammological Center, Loginov Moscow Clinical Scientific and Practical Center, Moscow, Russia; ^7^ Department of Healthcare Promotion, National Research Center for Therapy and Preventive Medicine, Moscow, Russia; ^8^ Department of Therapy, Clinical Pharmacology and Emergency Medicine, A.I. Yevdokimov Moscow State University of Medicine and Dentistry, Moscow, Russia; ^9^ M.I. Perelman Department of Phthisiopulmonology and Thoracic Surgery, I.M. Sechenov First Moscow State Medical University (Sechenov University), Moscow, Russia; ^10^ Department of English Language, Institute of World Economy, Diplomatic Academy of the Russian Foreign Ministry, Moscow, Russia; ^11^ Department of Scientific Support for Laboratory Research of Products and Environment Objects, F.F. Erisman Federal Scientific Center of Hygiene of Federal Service for Surveillance on Consumer Rights Protection and Human Wellbeing, Moscow Region, Mytishchi, Russia

**Keywords:** diphtheria, tetanus, DTP vaccine, immunity, pregnant women, healthcare professionals, pertussis

## Abstract

Despite more than 50 years of primary immunization against diphtheria, pertussis, and tetanus in Russia, complicated illnesses, including fatal ones, still occur. The goal of this preliminary cross-sectional study is to see how well pregnant women and healthcare workers are protected against diphtheria, pertussis, and tetanus. The required sample size (pregnant women and healthcare professionals, as well as pregnant women of two age categories) for this preliminary cross-sectional study was calculated using a confidence value of 0.95 and a probability of 0.05. The required number of participants in each group calculated sample size must be at least 59 people. In the Moscow region (Solnechnogorsk city, Russia), a cross-sectional study of pregnant patients and healthcare professionals interacting with children regularly as part of their job from numerous medical organizations was conducted in the year 2021 (*n* = 655). Antibodies to diphtheria, tetanus, and pertussis toxoids and microorganisms were measured using an enzyme-linked immunosorbent assay (ELISA). The STATISTICA and IBM SPSS Statistics 26.0 were used to process the study results statistically. Descriptive statistics methods, the Mann–Whitney *U*-test, discriminant analysis with the stepwise selection and analysis of ROC-curves were applied. IgG against diphtheria was found in 99.5% of pregnant women, tetanus in 91.5%, and pertussis in only 36.5%. According to the results of the discriminant analysis, the value of IgG to pertussis is linked to the value of IgA to pertussis and the gestational periods. Immunity to diphtheria was discovered in 99.1% of medical personnel, tetanus in 96.9%, and pertussis in 43.9%, no significant variations with age. When comparing the levels of immunity of pregnant women and healthcare professionals, it was shown that healthcare workers have greater levels of immunity against diphtheria and tetanus. The novel contribution of this study is that it will reveal the proportion of those vulnerable to pertussis, diphtheria, and tetanus among health workers and pregnant women in all age groups under the current national immunization program in Russia. Considering the data obtained from the preliminary cross-sectional study, we believe that it is necessary to conduct a full-scale study on a larger sample and, based on that, make certain changes to the national immunization program in Russia.

## Introduction

Despite many years of sequential immunization, pertussis, diphtheria, and tetanus remain important diseases for healthcare in all countries of the world (1). The ongoing registration of severe forms of these diseases and mortality makes this topic relevant. Since the late 1950s, Russia has used an adsorbed diphtheria-tetanus-pertussis (DTP) vaccine to prevent diphtheria, tetanus, and pertussis in the general population. The primary immunization against diphtheria, tetanus, and pertussis in Russia begins with three vaccinations at 3, 4.5, and 6 months of age (1). In this case, both vaccines containing a whole-cell pertussis component (DTP vaccine) and those with a cell-free pertussis component (Pentaxim, Infanrix, Infanrix Hexa) are used, at the discretion of parents or on the recommendations of medical workers. As part of this immunization strategy, vaccination coverage of children at the age of 12 months has not fallen below 95% of all children in the Russian Federation eligible for vaccination at the appropriate age since 2005 (2).

Regarding booster vaccinations, Russia adheres to the principle of world medical practice and recommends revaccination against diphtheria and tetanus in school, adolescence, and adulthood (at age 14 and with a frequency of every 10 years) (1). At the same time, for healthcare professionals, such revaccination is mandatory and serves as a criterion for admission to the profession.

On the other hand, existing pertussis booster vaccination regimens differ greatly between nations. Unlike diphtheria and tetanus, the Russian Federation's current national immunization calendar allows for just one booster vaccine against pertussis (at 18 months of age) and does not allow for additional booster vaccinations (1). Booster immunization of schoolchildren and adults with a vaccine with a whole-cell pertussis component was canceled in Russia in 1980. The main national strategy was the protection of children under one year of age and the impact of post-infection immunity formed at school age on the protection of the population. The first (and currently the only) vaccine preparation with an acellular pertussis component for revaccination of children over 4 years of age was registered in the Russian Federation only in 2016 (ADACEL Tdap vaccine – Tetanus Toxoid, Reduced Diphtheria Toxoid and Acellular Pertussis Vaccine Adsorbed). However, there is no similar Russian-made national vaccine for the immunization of adolescents, and discussions are underway about the cost-effectiveness of including their booster immunization in the Russian Federation's current national immunization calendar. According to the recommendations of healthcare professionals in Russia, parents can independently vaccinate school-age children in private medical centers with the ADACEL Tdap vaccine at their own expense.

This is different from a number of pertussis vaccination strategies used in other countries. Booster vaccinations against diphtheria and tetanus also include an obligatory pertussis (typically acellular) component in several countries. Revaccination of pregnant women against pertussis is available in the United States, the United Kingdom, and Australia (3, 4). Booster immunization of medical personnel working with children has been established in some European countries like Denmark, the Netherlands, and Finland (5, 6). The United States is one of those countries where the diphtheria-tetanus-pertussis vaccination regulations are comprehensive. Thus, in accordance with the recommendations of the Centers for Disease Control and Prevention (CDC), vaccination with Td or Tdap is recommended every 10 years for the entire adult population of the country, including healthcare professionals, and since 2012, it has been recommended that pregnant women be vaccinated in each pregnancy optimally from 27 to 36 weeks of gestation (7, 8).

Because of the high coverage of booster vaccines among persons in the designated categories and the excellent immunogenicity of diphtheria and tetanus toxoid, the incidence of diphtheria and tetanus has been reduced to a single occurrence per year in most countries throughout the world. With such low occurrences, monitoring of the level of particular immunity to certain diseases becomes the most objective technique for identifying susceptible populations.

Despite years of widespread vaccination against pertussis, such a significant and long-lasting reduction in incidence rates has not been accomplished. Children in their first year of life are still the most vulnerable to serious complications and mortality from pertussis all around the world. Every year in Russia, between 1,271 and 2,443 children under the age of one become ill (9–11). Since 2017, in Russia, pertussis fatalities have been reported in this age group practically every year. All ill children were, on average, either not vaccinated (due to age or parental refusal) or were only partially vaccinated and received less than three components throughout the vaccine cycle (10).

Children under the age of 12 months are not highly sociable, and their interactions are frequently confined to family members and healthcare professionals. That is why we consider these people to be possible infection sources and monitor their immune state in respect to these three diseases.

Nosocomial pertussis from healthcare professionals has been investigated in Australia, Europe, and the United States. Outbreaks of this disease have been reported, with over 5 persons infected, with the source of infection for children under the age of one year being workers of pediatric or maternity units (12).

Healthcare professionals are at the forefront of dealing with many infectious agents, that is why it is necessary to have an acceptable degree of protection for this occupational risk category. This is vital for both the medical personnel and their patients.

The main mechanism for protecting infants under two months of age from many infections, including diphtheria, tetanus, and pertussis, is the transplacental transfer of maternal immunoglobulin G (IgG) antibodies (3, 9, 10, 13–17). It should also be mentioned that protection is also obtained *via* the infant's own primary immunizations. It is a combination of maternal antibodies and the primary immunization series that allows for protection of infants under one year of age. This method, however, can only be effective if pregnant women have high levels of antibodies.

Some Russian experts (9, 10) are disputing whether pertussis booster doses should be included in the Russian Federation's current national immunization calendar for healthcare professionals and pregnant women. There are, however, few scientific studies dedicated to assessing and describing the level of specific immunity to pertussis among high-risk populations such as medical personnel and pregnant women, and they all ignore the condition of immunity to diphtheria and tetanus. Traditionally, researchers have used specific IgG-class antibodies to assess seroprevalence to these infections, the absence of which characterizes vulnerability to diseases and, as a result, the risk of being a source of infection (9, 13, 18). An interesting criterion for evaluating post-infection immunity to pertussis, in our opinion, is the presence of specific antibodies of the IgA class, which can appear only upon direct contact with *Bardetella pertussis* and show the presence of a hidden epidemic process.

The aim of this preliminary cross-sectional study was to determine the immunity to diphtheria, tetanus, and pertussis among risk groups thought to be a sources of infection for infants under the age of one.

## Materials and methods

In the Moscow region (Solnechnogorsk city, Russia), a preliminary cross-sectional study of pregnant patients and healthcare professionals from numerous medical organizations was conducted. The study included healthcare personnel interacting with children regularly as part of their job (providing medical care to women during pregnancy and childbirth; having contact with a newborn or working with children in hospitals). The study was conducted in accordance with the Declaration of Helsinki, and approved by the Local Ethic Committee of the I.M. Sechenov First Moscow State Medical University (Sechenov University) (protocol №13–22, 22 June 2022). Informed consent was obtained from all subjects involved in the study.

The required sample size (pregnant women and healthcare professionals, as well as pregnant women of two age categories) for this preliminary cross-sectional study was calculated using an online calcullated formula, as described by Viechtbauer W. et al. (19). Assumptions were made for a confidence value of 0.95 and a probability (*p*-value) of 0.05. The required number of participants in each group calculated sample size must be at least 59 people.

Blood Serum was taken from the study participants for further antibody analysis. The study was completed in the year 2021. All subjects were in good health at the time of the study and had no previous history of pertussis, diphtheria, tetanus, or contact with a sick person during the last 12 months. A total of 655 blood samples were examined, with 200 coming from pregnant women aged 18 to 39 and 455 from healthcare professionals aged 18 to 59. There were 114 (57.0%) pregnant women in the first trimester, 45 subjects (22.5%) in the second trimester, and 41 subjects in the third trimester (20.5%). The test for immunoglobulin A (IgA) antibodies to pertussis (anti-Bordetella pertussis toxin IgA; Anti-Bordetella pertussis toxin (IgA) kits from EUROIMMUN AG (Germany)) was performed on 114 blood samples from pregnant women and 100 blood samples from medical personnel. Detecting anti-Bordetella pertussis toxin IgA levels was used to tentatively assess the possibility of recent (within the last 12 months) pertussis, in individuals with no history of this disease, which can be a source of infection for infants under one year of age.

Antibodies to diphtheria, tetanus, and pertussis toxoids and microorganisms were measured using enzyme-linked immunosorbent assay (ELISA)—the Anti-Diphtheria Toxoid ELISA (IgG), Anti-Tetanus Toxoid ELISA (IgG), and Anti-Bordetella pertussis toxin (IgA) kits from EUROIMMUN AG (Germany) and RIDASCREEN Bordetell IgG. The findings of the study were evaluated using instructions provided by the test system manufacturers in line with WHO standards. IgG to diphtheria toxoid > 0.01 IU/ml, tetanus toxoid > 0.1 IU/ml, and pertussis microorganism > 14 U/ml (when determining IgG) and > 12 IU (when determining IgA in the test system Anti-Bordetella pertussis toxin) were all regarded positive. Intraassay and interassay coefficients were prescribed in the instructions for the test system and amounted to a coefficient of variation (CV) using 3 samples. The intra-assay CVs are based on 20 determinations, and the inter-assay CVs are based on 4 determinations performed in 6 different test runs.

The following gradations were used to assess the levels of antibodies in seropositive persons. For diphtheria antibodies, low levels are 0.01–0.099 IU/ml, medium levels are 0.1–1.0 IU/ml, and high levels are >1.0 IU/ml. For tetanus antibodies, low levels are 0.1–0.5 IU/ml, average levels are 0.6–1.1 IU/ml, and high levels are >1.1 IU/ml. For pertussis infection, 14–18 U/ml indicates very low levels of antibodies, 19–30 U/ml indicates low levels, 31–50 U/ml indicates medium levels, and >50 U/ml indicates high levels of antibodies.

Pregnant women in two age groups were studied: 18–29 years old (100 subjects) and 30–39 years old (100 subjects). The following age categories were considered among healthcare professionals: 109 subjects between the ages of 18 and 29, 153 subjects between the ages of 30 and 39, 94 subjects between the ages of 40 and 49, and 99 subjects between the ages of 50 and older.

The STATISTICA Base statistical software tool and IBM SPSS Statistics 26.0 were used to process the study results statistically. Regarding the observed groupings, the normality test revealed a significantly asymmetric distribution. In the ordered sample, absolutes, medians (M), and interquartile ranges (IQR) were calculated. The relative values were presented as absolutes, percentages (%), and 95% Clopper-Pearson confidence intervals (95% CI). The Mann–Whitney *U*-test was chosen to check the statistical hypotheses of the difference between the compared groups by age, antibody level, and test result (positive or negative) due to the comparable nature of the distribution.

According to the null hypothesis, there were no differences between the compared groups. When testing statistical hypotheses, the crucial value of the significance level (*p*) was set at *p* ≤ 0.01. The probability of having protective levels of IgG antibodies to the pertussis microbe in pregnant women was determined using a discriminant analysis with the stepwise selection method to determine the dependence of the probability of having protective levels of anti-pertussis IgG on age of pregnant women (years), gestational age (weeks), the levels of anti-diphtheria IgG, the levels of anti-tetanus IgG, and the levels of anti-pertussis IgA. The quality of the predictive discriminant model was assessed using the analysis of the ROC-curve, the area under the ROC-curve (AUC) with 95% confidence interval (95% CI) and the level of statistical significance (*p*-value).

## Limitations of the study

In this study, there were no pregnant women over the age of 39 or healthcare professionals over the age of 59.

## Results

Analysis of the data obtained showed that 199 (99.5%; 95% CI: 97.2–99.9%) pregnant women had specific IgG antibodies to diphtheria toxoid (Table 1). At the same time, in 68.3% of them, the levels of IgG antibodies were assessed as “high” (>1.0 IU/ml).

**Table 1:** IgG blood serum levels to diphtheria, tetanus, and pertussis in pregnant women.

Availability IgG to:	Age group (years)	Number of examined	Of them			
			Seropositive		Seronegative	
			*N* (subjects)	% (95% CI)	*N* (subjects)	% (95% CI)
Diphtheria toxoid	18–29	100	99	99.0% (94.55–99.98%)	1	1.0% (0.03–5.45%)
	30–39	100	100	100.0% (96.38–100.00%)	0	0.0% (0.00–3.62%)
**Total**	**18–39**	**200**	**199**	**99.5%** **(97.25–99.99%)**	**1**	**0.5%** **(0.01–2.75%)**
Pertussis toxin and filamentous hemagglutinin	18–29	100	39	39.0% (29.40–49.27%)	61	61.0% (50.73–70.60%)
	30–39	100	34	34.0% (24.82–44.15%)	66	66.0% (55.85–75.18%)
**Total**	**18–39**	**200**	**73**	**36.5%** **(29.82–43.58%)**	**127**	**63.5%** **(56.42–70.18%)**
Tetanus toxoid	18–29	100	91	91.0% (83.60–95.80%)	9	9.0% (4.20–16.40%)
	30–39	100	92	92.0% (84.84–96.48%)	8	8.0% (3.52–15.16%)
**Total**	**18–39**	**200**	**183**	**91.5%** **(86.74–94.97%)**	**17**	**8.5%** **(5.03–13.26%)**

IgG antibodies to tetanus toxoid were detected in 183 pregnant women (91.5%; 95%CI: 86.7–95.0%), of which 67.4% were rated as “high”.

At the same time, it was not possible to identify a significant difference between the proportion of those seropositive in the age groups of 18–29 years and 30–39 years for both diphtheria and tetanus: 99.0% (95% CI: 94.6%–99.98%) vs. 100.0% (95% CI: 96.4–100.0%) and 91.0% (95% CI: 83.6–95.8%) vs. 92.0% (95% CI: 84.8–96.5%), respectively (*p* ≤ 0.01).

Only 36.5% (95% CI: 29.8–43.6%) of pregnant women (73 subjects) had IgG antibodies to pertussis. At the same time, in most of them (87.6%), the levels of IgG antibodies were quite high (>26 U/ml), and in 22.5% this parameter was recorded in the range of 50 U/ml and above. The presence of IgA was registered in 14 (12.3%) out of 114 examined pregnant women (Table 2). Their levels varied widely, from 19.3 to 170 U/ml.

**Table 2:** IgA blood serum levels to pertussis in pregnant women.

Age group (years)	Number of examined	Of them			
		Seropositive		Seronegative	
		*N* (subjects)	% (95% CI)	*N* (subjects)	% (95% CI)
Total	114	14	12.3% (6.879–19.746)	100	87.7% (80.254–93.121)

In most of the surveyed pregnant women (57.0%), the gestational age corresponded to the first trimester of pregnancy (Table 3). The proportion of pregnant women with IgG antibodies to diphtheria toxin did not depend on the duration of pregnancy (*p* ≤ 0.01) and was high in almost all subjects, from 99.1% (95% CI: 95.2–99.9%) in the first trimester to 100.0% (95% CI: 91.4–100.0%) in the third trimester.

**Table 3:** IgG blood serum levels to diphtheria, tetanus, and pertussis in pregnant women at different stages of pregnancy.

Antigen	Trimester	Total	Of them			
			Seropositive		Seronegative	
			*N* (subjects)	% (95% CI)	*N* (subjects)	% (95% CI)
Diphtheria toxoid	I	114	113	99.1% (95.21–99.98%)	1	0.9% (0.02–4.79%)
	II	45	45	100.0% (92.13–100.00%)	0	0.0% (0.00–7.87%)
	III	41	41	100.0% (91.40–100.00%)	0	0.0% (0.00–8.60%)
**Total**	** **	**200**	**199**	**99.5%** **(97.25–99.99%)**	**1**	**0.5%** **(0.13–2.75%)**
Pertussis toxin and filamentous hemagglutinin	I	114	44	38.6% (29.63–48.17%)	70	61.4% (51.83–70.37%)
	II	45	14	31.1%18.17–46.65%)	28	62.2% (46.94–77.88%)
	III	41	13	31.7% (18.09–48.09%)	26	63.4% (46.94–77.88%)
**Total**	** **	**200**	**71**	**35.5%** **(28.88–42.56%)**	**124**	**62.0%** **(54.89–68.75%)**
Tetanus toxoid	I	114	107	93.9% (87.76–97.50%)	7	6.1% (2.50–12.24%)
	II	45	41	91.1% (78.78–97.53%)	4	8.9% (2.48–21.22%)
	III	41	35	85.4% (70.83–94.43%)	6	14.6% (5.57–29.17%)
**Total**	** **	**200**	**183**	**91.5%** **(86.74–94.97%)**	**17**	**8.5%** **(5.03–13.26%)**

The proportion of those seropositive for tetanus was lower, from 93.9% (95% CI: 87.8–97.5%) in the first trimester to 85.4% (95% CI: 70.8–94.4%) in the third, but it was also not possible to establish significant differences between the proportion of seronegative subjects at different stages of pregnancy (*p* ≤ 0.01).

Most seronegative, susceptible subjects were identified as having pertussis infection. At the same time, the proportion of those susceptible was equally high in all three groups, exceeding 60% (from 61.4% (95% CI: 51.8–70.4%)—women in the first trimester of pregnancy to 63.4% (95% CI: 46.9–77.9%) of women in the third trimester of pregnancy).

Considering the previously identified specific IgA antibodies to pertussis (Table 2), further studies were conducted to examine a possible relationship between their presence and the presence of high IgG values. As a result of discriminant analysis, the following novel model was obtained, which makes it possible to predict the probability of detecting IgG in pertussis depending on the gestation period (weeks) and the levels of IgA:

YIgG=0.493+0.051×XIgA−0.056×Xw

where YIgG is a discriminant function characterizing the probability of having IgG antibodies to pertussis, XIgA are the levels of IgA antibodies to pertussis (IU/ml), Xw is the gestation period (weeks).

**Figure 1:**
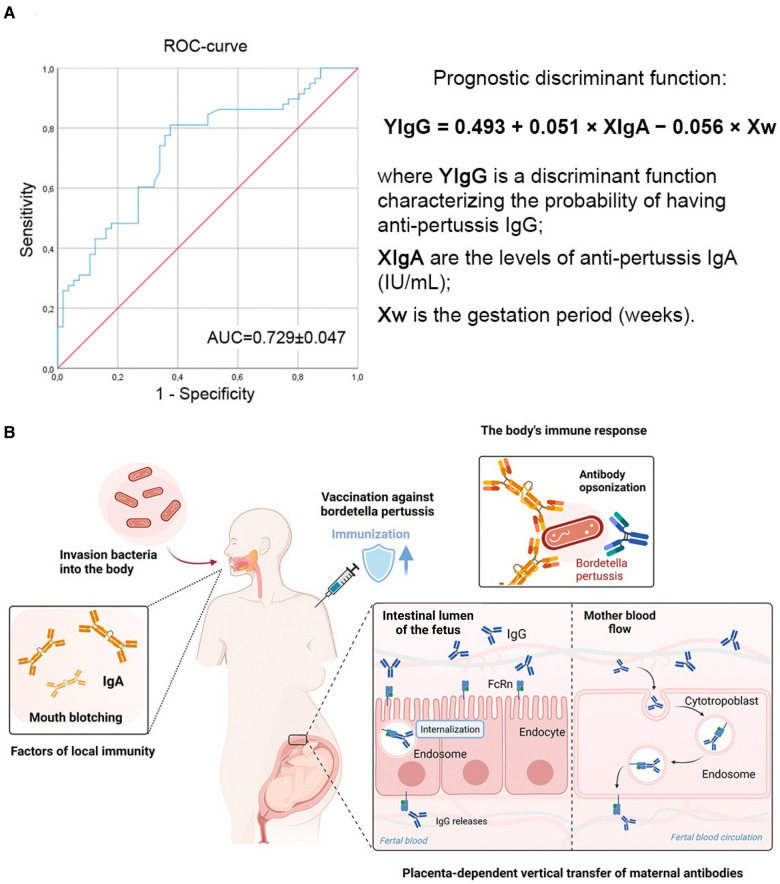
The presence of IgG to the pertussis pathogen depends on the levels of IgA and gestation period (weeks). (**A**) ROC-curve characterizing the dependence of the probability of having a protective level of anti-pertussis IgG on the values of the prognostic discriminant function. The area under the curve (AUC) was 0.729 ± 0.047 with 95% CI: 0.63–0.822 (*p* < 0.001). (**B**) Circulation of specific anti-pertussis IgA and IgG in pregnant women. Infant immunity against Bordetella pertussis is advanced by the placenta-dependent vertical transfer of maternal antibodies. Anti-pertussis IgA are produced together with anti-pertussis IgG after encounter with the *Bordetella pertussis*, but not during DTP vaccination.

The discrimination constant dividing the subjects into two groups was determined as the value of the function equidistant from the centroids, which was −0.376 in the group with no IgG antibodies to pertussis, and in the presence of it was 0.363. Accordingly, the discrimination constant is −0.0065 (Figure 1).

When comparing the mean values of the discriminant function in both groups using the Wilks coefficient *λ*, statistically significant differences were established (*p* < 0.001).

The belonging of pregnant women to the groups of high or low probability of having IgG antibodies to pertussis was determined based on the calculated values of the prognostic discriminant function: if the value of the function was more than −0.0065, the pregnant woman belonged to the group with a high probability of having a protective levels of IgG antibodies to pertussis; if the value of the function was less than −0.0065 the pregnant woman belonged to the low probability group.

The sensitivity of the model was 75.9%, the specificity was 64.3%, and the overall diagnostic significance was 70.2%.

In our further studies, the state of humoral immunity to diphtheria, tetanus, and pertussis was assessed among healthcare professionals. According to the Russian Federation's current national immunization calendar, healthcare professionals in Russia should receive booster immunizations against diphtheria and tetanus at age 14 and every 10 years thereafter. Booster immunization against diphtheria, tetanus, and pertussis is a requirement for entry and employment in the medical profession (1).

It was found that specific IgG antibodies to diphtheria toxoid were detected in 99.1% (95% CI: 97.8–99.8%) of the samples examined. At the same time, the proportion of subjects immune against diphtheria was quite high in all age groups, and ranged from 97.0% (95% CI: 91.4–99.4%) (age group 50–59 years) to 100% (95% CI: 96.2–100.0%) (40–49 years) and 100% (95% CI: 97.6–100.0) (30–39 years old) (Table 4).

**Table 4:** IgG blood serum levels to diphtheria in healthcare professionals.

№	Age group (years)	Number of examined	Of them			
			Seropositive		Seronegative	
			*N* (subjects)	% (95% CI)	*N* (subjects)	% (95% CI)
1	1–29	109	108	99.1% (94.99–99.98%)	1	0.9% (0.02–5.01%)
2	30–39	153	153	100% (97.62–100.00%)	0	0.0% (0.00–2.38%)
3	40–49	94	94	100% (96.15–100.00%)	0	0.0% (0.00–3.85%)
4	50–59	99	96	97.0% (91.40–99.37%)	3	3.0% (0.63–8.60%)
**5**	**Total**	**455**	**451**	99.1% (97.76–99.76%)	4	0.9% (0.24–2.24%)

**Figure 2:**
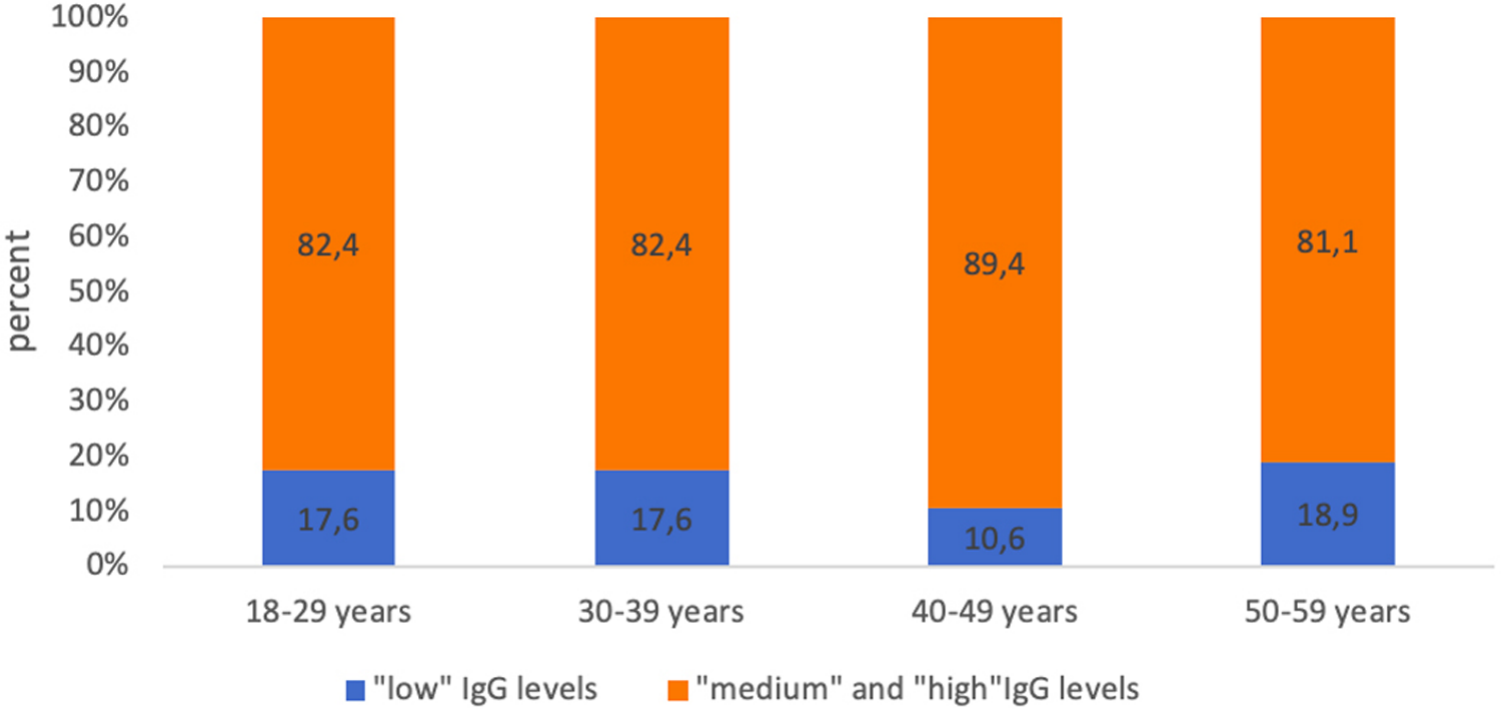
Proportion of seropositive healthcare professionals with “low” anti-diphtheria IgG levels and with “medium” and “high” anti-diphtheria IgG levels in different age groups.

At the same time, the analysis of the levels of IgG antibodies showed that in more than 70% of the examined in each age group was assessed as “high” (>1.0 IU/ml). The proportion of subjects with “low” levels (0.01–0.099 IU/ml) of IgG antibodies ranged from 12.8% among healthcare professionals aged 40–49 years to 26.0% among the age group 50 years and older (Figure 2).

**Figure 3:**
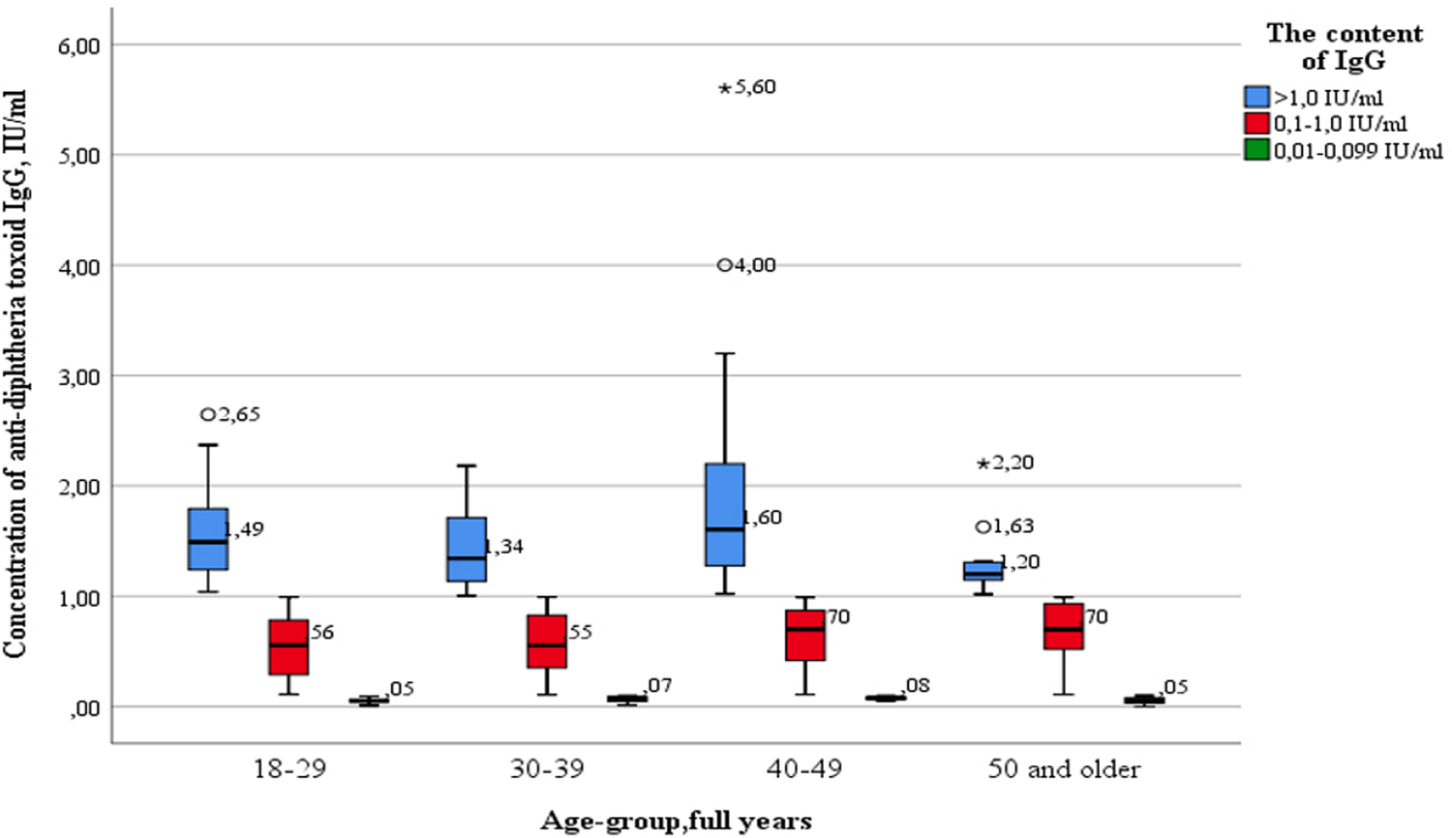
The levels of diphtheria immunity in different age groups of healthcare professionals.

Simultaneously, no statistically significant differences were found between age groups, both when comparing healthcare professionals of different ages with “low” levels of IgG antibodies and when comparing subjects with “medium” and “high” levels of IgG antibodies (Figure 3).

Outliers were observed in three age groups: 18–29 years old (2.65 IU/ml—significant), 40–49 years old (4.00 IU/ml—significant) and 50 years and older (1.63 IU/ml—significant; 2.20 IU/ml—insignificant). The data is grouped asymmetrically, the greatest asymmetry is observed in the age groups of 18–29, 30–39 and 40–49 years among subjects with “high” levels of IgG antibodies (>1.0 IU/ml). In the age groups of 18–29, 30–39, 40–49 years, there is a relationship between levels of IgG antibodies and the grouping density: the lower the content, the denser the grouped data. In the age group of 50 years and older, the distribution density increases in the appropriate order: healthcare professionals with “medium” (0.1–1.0 IU/ml), “high” (>1.0 IU/ml), and “low” (0.01–0.099 IU/ml) antibody levels. The greatest dispersion is observed in subjects of the age group 40–49 years old, with “high” levels of IgG. The smallest in the same age group with “low” levels of IgG antibodies.

The proportion of healthcare professionals IgG levels to tetanus infection also met the criterion of epidemiological well-being adopted in Russia (more than 95% of immune subjects), amounting to 96.9% (95% CI 94.9–98.3%). By age group, the proportion protected from tetanus ranged from 89.9% (95% CI: 82.2–95.0%) (50–59 years) to 99.1% (95% CI: 95.0–99.98%) (18–29 years) (Table 5).

**Table 5:** IgG blood serum levels to tetanus in healthcare professionals.

№	Age group (years)	Number of examined	Of them			
			Seropositive		Seronegative	
			*N* (subjects)	% (95% CI)	*N* (subjects)	% (95% CI)
1	18–29	109	108	99.1% (94.99–99.98%)	1	0.9% (0.02–5.01%)
2	30–39	153	151	98.7% (95.36–99.84%)	2	1.3% (0.16–4.64%)
3	40–49	94	93	98.9% (94.22–99.97%)	1	1.1% (0.03–5.79%)
4	50–59	99	89	89.9% (82.21–95.05%)	10	10.1% (4.95–17.79%)
**5**	**Total**	**455**	**441**	96.9% (94.89–98.31%)	14	3.1% (1.69–5.11%)

**Figure 4:**
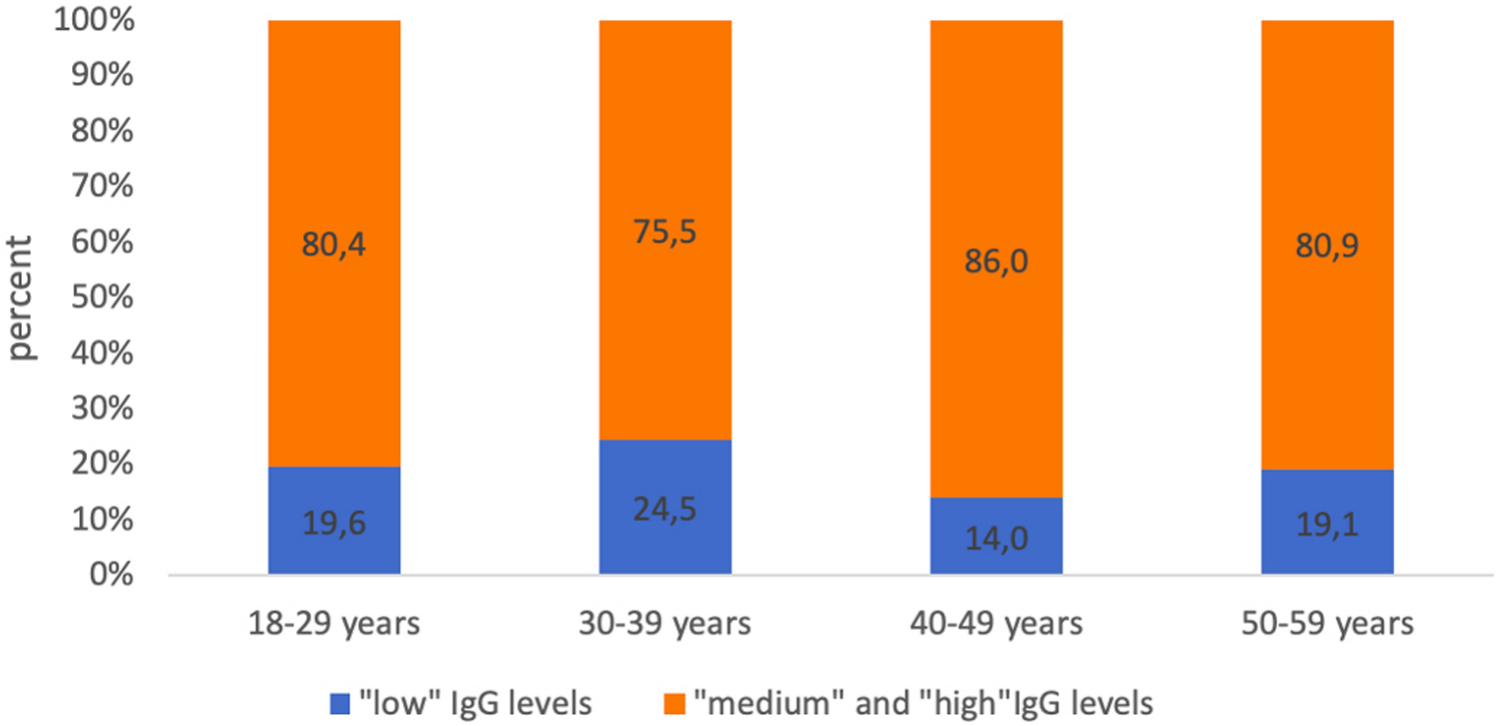
Proportion of seropositive healthcare professionals with “low” anti-tetanus IgG levels and with “medium” and “high” anti-tetanus IgG levels in different age groups.

The proportion of subjects who had “low” levels (0.1–0.5 IU/ml) of IgG antibodies to tetanus ranged from 19.6% (18–29 years) to 24.5% (30–39 years) (Figure 4).

**Figure 5:**
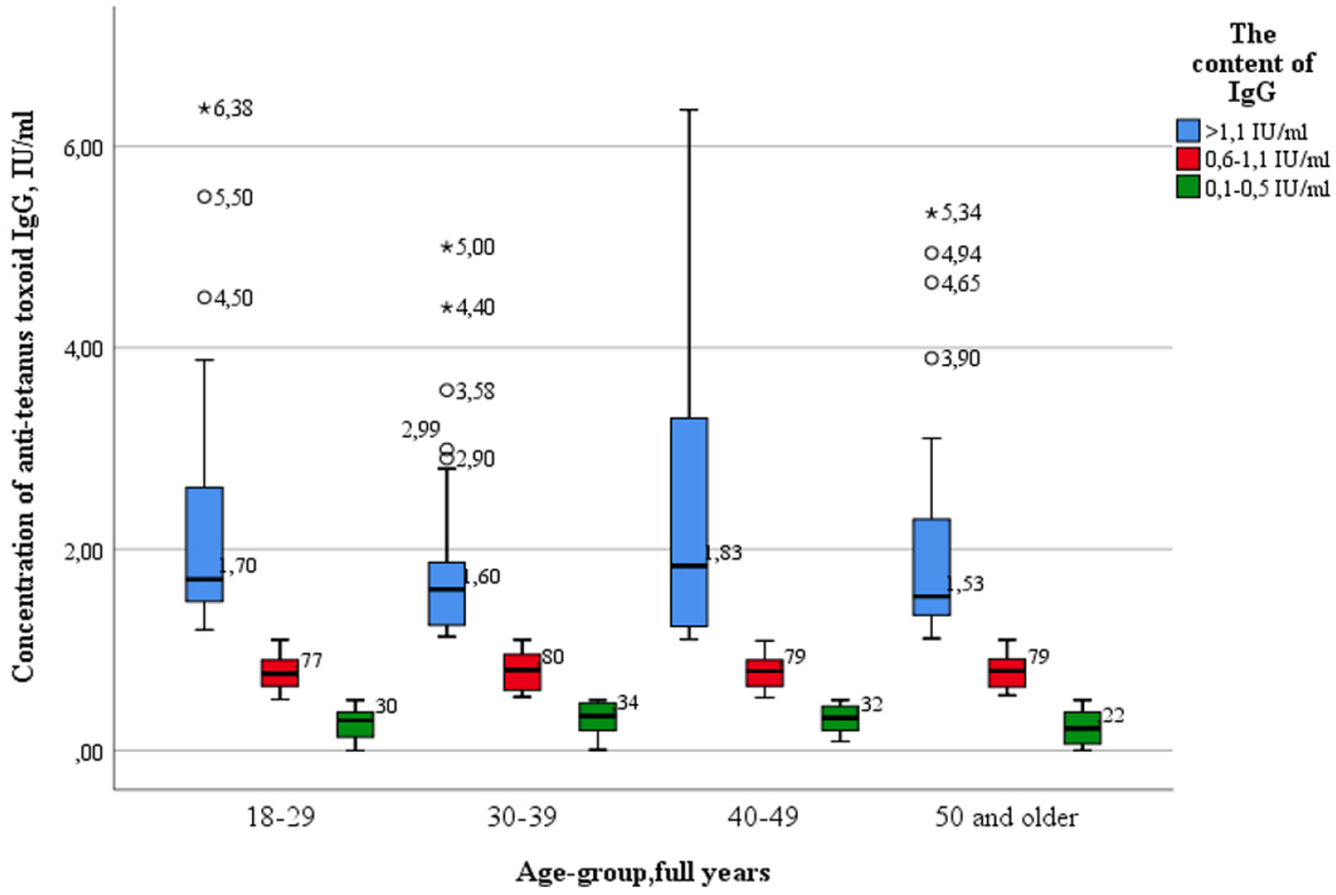
The level of tetanus immunity in different age groups of healthcare professionals.

Also, as in the case of diphtheria infection, there were no statistically significant differences between the age groups of subjects with “high levels” of IgG antibodies (>1.1 IU/ml) and those with “low” (0.1–0.5 IU/ml) and “medium” levels of IgG antibodies (0.6–1.1 IU/ml) when comparing the concentrations of IgG antibodies by age groups (Figure 5).

Key values: medians in the age group 18–29 years old were 0.39 IU/ml; 0.80 IU/ml; 1.70 IU/ml, in the group—30–39 years 0.40 IU/ml; 0.80 IU/ml; 1.60 IU/ml, in the group 40–49 years—0.44 IU/ml; 0.80 IU/ml; 1.83 IU/ml, in the group of 50 years and older—0.39 IU/ml; 0.83 IU/ml and 1.53 IU/ml. Outliers were observed in three age groups: 18–29 years old (4.50 IU/ml; 5.50 IU/ml—significant, 6.38 IU/ml—insignificant), 30–39 years old (2.90 IU/ml; 2.99 IU/ml; 3.58 IU/ml—significant, 4.40 IU/ml; 5.00 IU/ml—insignificant) and 50 years and older (3.90 IU/ml; 4.65 IU/ml;4.94 IU/ml—significant; 5.34 IU/ml—insignificant). The data is grouped asymmetrically. The greatest asymmetry is observed in the age groups of 18–29 years and 40–49 years, among subjects with “high” levels of IgG antibodies. In all age groups, the lowest distribution density and the highest dispersion occur among subjects with “high” levels of IgG antibodies.

For the causative agent of pertussis, IgG antibodies were detected only in 43.96% (95% CI: 39.3–48.7%) of the blood serum of healthcare professionals. Accordingly, 56.04% (95% CI 51.3–60.6%) of healthcare professionals were seronegative for this infection. In the context of age groups, this parameter fluctuated from 49.5% (95% CI: 39.3–59.7%) (50–59 years old) to 65.4% (95% CI: 57.2–72.8%) (30–39 years old) (Table 6).

**Table 6:** IgG blood serum levels to pertussis in healthcare professionals.

№	Age group (years)	Number of examined	Of them			
			Seropositive		Seronegative	
			*N* (subjects)	% (95% CI)	*N* (subjects)	% (95% CI)
1	18–29	109	50	45.9% (36.29–55.69%)	59	54.1% (44.32–63.71%)
2	30–39	153	53	34.6% (27.14–42.75%)	100	65.4% (57.25–72.86%)
3	40–49	94	47	50.0% (39.51–60.49%)	47	50.0% (39.51–60.49%)
4	50–59	99	50	50.5% (40.27–60.71%)	49	49.5% (39.29–59.73%)
**5**	**Total**	**455**	**200**	43.96% (39.34–48.65%)	255	56.04% (51.35–60.66%)

**Figure 6:**
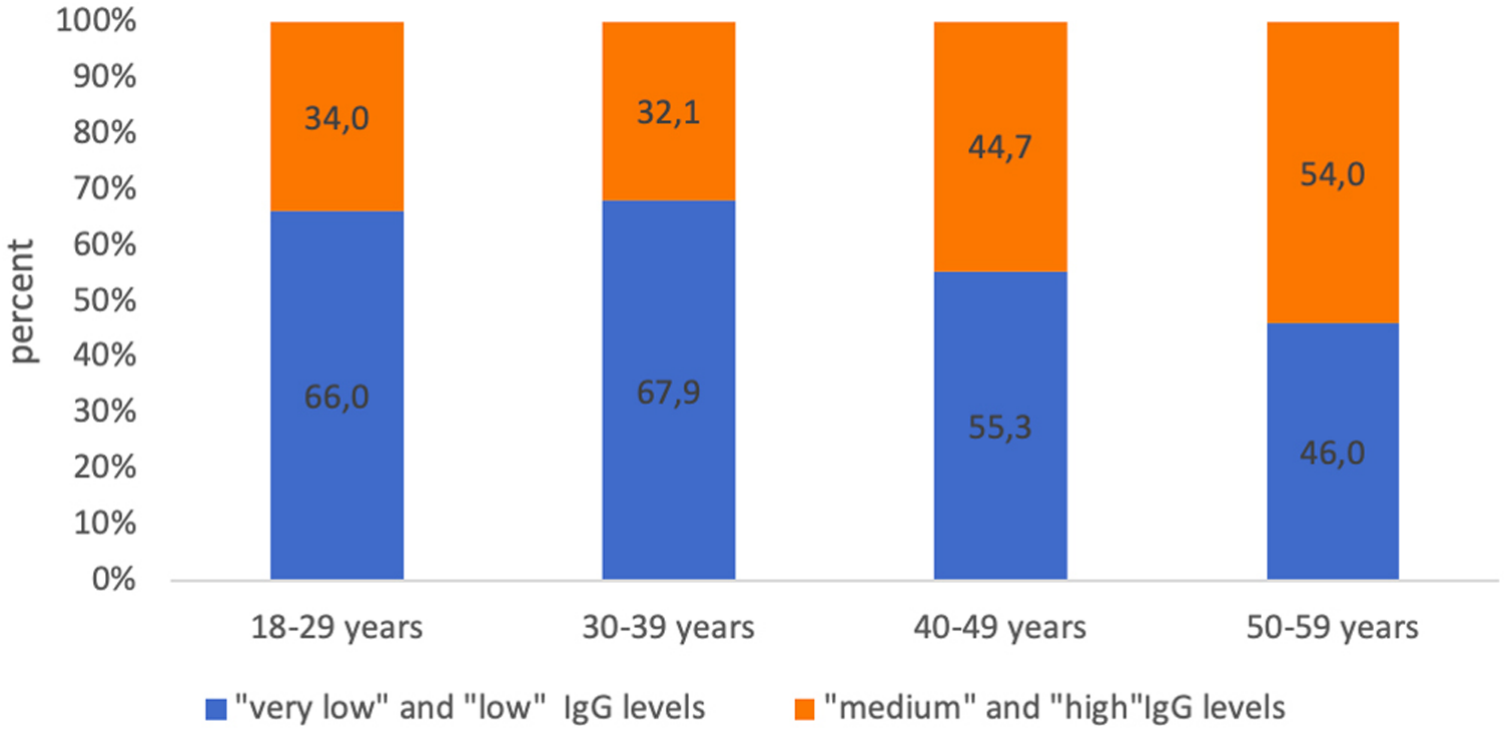
Proportion of seropositive healthcare professionals with “low” anti-pertussis IgG levels and with “medium” and “high” anti-pertussis IgG levels in different age groups.

With a seronegative proportion of more than 30% in each age group, the high proportion of seropositive subjects with “low” and “very low” levels of IgG antibodies attracts attention. This indicator fluctuated in each age group from 46.0% (50–59 years old) to 66.0% (age group 18–29 years old) (Figure 6).

**Figure 7:**
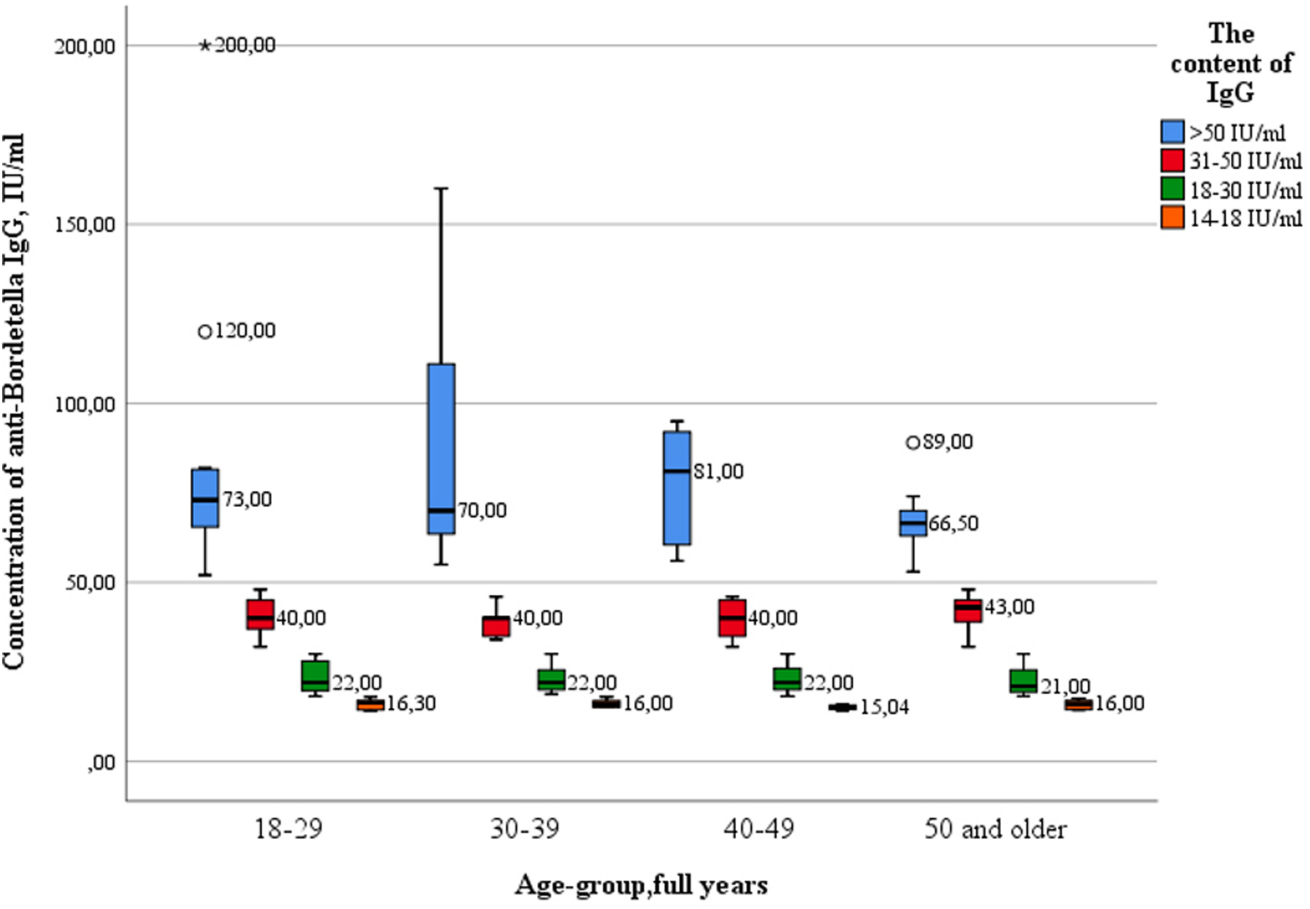
The level of pertussis immunity in different age groups of healthcare professionals.

The analysis showed that there were no statistically significant differences in the concentration of IgG between the age groups of healthcare professionals (Figure 7). The greatest variance in the parameter of the value of IgG antibodies was observed in subjects with “high” levels of IgG antibodies in the age group of 30–39 years.

Key values: medians in the 18–29 age group were 16.30 IU/ml; 22.0 IU/ml; 40.0 IU/ml; 73.0 IU/ml, in the group of 30–39 years 16.0 IU/ml; 22.0 IU/ml; 40.0 IU/ml; 70.0 IU/ml, in the group of 40–49 years old—15.04 IU/ml; 22.00 IU/ml; 40.00 IU/ml; 81.0 IU/ml, in the group of 50 years and older—16.0 IU/ml, 21.0 IU/ml, 43.0 IU/ml and 66.50 IU/ml. Outliers were observed in two age groups: 18–29 years old (120.0 IU/ml—significant), and 50 years and older (89.0 IU/ml—significant). The data is grouped asymmetrically. The largest asymmetry is observed in the age group of 40–49 years among subjects with “high” levels of antibodies. In the age groups of 18–29, 30–39 and 40–49 years, the largest interquartile range is observed among groups with “high” levels of antibodies. The greatest dispersion is observed in subjects in the age group of 30–39 years, with “high” levels of IgG. The lowest was in the age group of 40–49 years, with “very low” levels of antibodies. The presence of IgA antibodies to pertussis was registered in 3 healthcare professionals out of 100 (3%) (Table 7).

**Table 7:** IgA blood serum levels to pertussis in healthcare professionals.

Age group (years)	Number of examined	Of them			
		Seropositive		Seronegative	
		*N* (subjects)	% (95% CI)	*N* (subjects)	% (95% CI)
Total	100	3	3.0% (0.623–8.518%)	97	97.0% (91.482–99.377%)

In this study, for the first time, a comparative analysis of the protective levels of IgG and IgA antibodies to the studied infections in the two studied risk groups in the Moscow region (Solnechnogorsk city) of the Russian Federation was carried out. In the final comparison of the pregnant women and healthcare professionals, statistically significant differences were obtained in the following serum values: the levels of IgG to diphtheria (*p* < 0.001) and tetanus (*p* < 0.001) were significantly higher in the group of healthcare professionals compared to pregnant women (Table 8). In pregnant women, a statistically significant excess of IgA levels for pertussis was revealed compared to healthcare professionals (*p* < 0.001).

**Table 8:** IgG blood serum levels to diphtheria, tetanus, and pertussis in pregnant women and healthcare professionals.

Serum study		Pregnant (*n* = 200)	Healthcare professionals (*n* = 455)	Mann–Whitney *U*-test (*p* two-sided)
Diphtheria toxoid	IgG, M [IQR]	0.38 [0.086–0.54525]	0.68 [0.1345–0.956]	*p* = 0.00001*
	Seronegative (abs.)	0	4	*p* = 0.61
Tetanus toxoid	IgG, M [IQR]	0.92 [0.409–0.97325]	1.09 [0.579–1.265]	*p* = 0.00304*
	Seronegative (abs.)	17	14	*p* = 0.00263*
Pertussis toxin and filamentous hemagglutinin	IgG, M [IQR]	19.76 [3.95–22.0]	18.13 [5.0–22.0]	*p* = 0.76181
	Seronegative (abs.)	127	255	*p* = 0.0748

* Statistically significant level *p* < 0.001.

## Discussion

The preliminary study data obtained indicates a high level of protection of the subjects from the indicated risk groups from diphtheria (99.5% (95% CI: 97.2–99.9%) of protected subjects among the examined cohort of pregnant women and 99.1% (95% CI: 97.8–99.8%) of protected subjects among the examined cohort of healthcare professionals) and tetanus (91.5% (95% CI: 86.7–95.0%) and 96.9% (95% CI: 94.9–98.3%) of protected subjects, respectively). This is comparable to the results obtained by researchers in other countries when testing for diphtheria and tetanus seroprevalence in the same risk groups (20, 21), or among adults, regardless of their profession (22, 23).

Also, the results obtained indicate high vaccination coverage against these infections in Russia and are the result of the ongoing immunization tactics (revaccination of the adult population against diphtheria and tetanus every 10 years and tracking immunity to diphtheria and tetanus in routine epidemiological surveillance).

Analysis of the results of a serological test for the presence of antibodies to diphtheria and tetanus in the blood serum of pregnant women in the considered age groups (18–29 years and 30–39 years) showed their comparability and closeness to the average parameter obtained for the entire sample. Analysis of the obtained results did not reveal differences in the levels of immunity of pregnant women in different age groups (*p* ≤ 0.01).

Regarding healthcare professionals, a slight decrease in the proportion of subjects with immunity to tetanus in the group of 50–59 years was shown (89.9% (95% CI: 82.2–95.0%) vs. 99.1% (95% CI: 95.0–99.9%) in the age group of 18–29 years). When screened for diphtheria, IgG levels remained high in all age groups, with only slight fluctuations. However, in recent years, authors, both in Russia and other countries, have noted a gradual loss of antidiphtheria immunity with age (17, 24–27). In many countries, an increase in the proportion of people aged 50 years and older who are seronegative to diphtheria toxin has been recorded (20). Perhaps the absence of such a trend in our study is due to the greater attention to the level of protection and the timing of immunization among healthcare professionals.

An analysis of levels of IgG antibodies against diphtheria and tetanus toxoid in the blood showed that more than 70% of healthcare professionals have “medium” (0.1–1.0 IU/ml and 0.6–1.1 IU/ml, respectively) and “high” (>1.0 IU/ml and >1.1 IU/ml) antibody levels. These values significantly prevailed within each age group, and there was no statistically significant difference between the ages. Comparable results were obtained in Catalonia, Spain in 2020. The proportion of individuals with protective levels of anti-tetanus IgG antibodies was estimated to be 94.7% (95% CI: 92.3–96.4%) of all ages of healthcare professionals and 85.1% (95% CI: 74.5–92.0%) of healthcare professionals aged 55 and older. The proportion of people in the same occupational group with IgG antibodies to diphtheria was less than in our studies and amounted to 68.6% (95% CI: 64.3–72.5%) among all age groups and 29.7% (95% CI: 19.9–41.6%) among those aged 55 and older (20). Perhaps, the discrepancy in the proportion of people with immunity to diphtheria may be explained by the mandatory state control over the timely immunization of medical workers in accordance with the Russian Federation's current national immunization calendar.

As for the proportion of subjects with immunity to pertussis, it was significantly lower among both pregnant women [36.5% (95% CI: 29.8–43.5%)] and healthcare professionals 43.96% (95% CI: 39.3–48.6%)).

Many researchers in various countries (28–31) have noted a relatively high proportion of people who are seronegative for pertussis (compared to the proportion of those who are seronegative for diphtheria and tetanus). For example, among healthcare workers in Mexico, the proportion of people with pertussis immunity was 18.3% (31). Researchers from Turkey examined medical workers from children's hospitals and found that a high proportion of them (39.5%) were susceptible to pertussis (29). The authors agree that people who are susceptible to this infection can infect children.

In our studies, the proportion of seronegative subjects did not significantly depend on the age of pregnant women (the parameter varied from 39.0% (95% CI: 29.4–49.2%) at the age of 18–39 years to 34.0% (95% CI: 24.8–44.1%) at the age of 30–39 years). Perhaps this result was influenced by the absence of pregnant women over the age of 39 in the study. At the same time, when comparing the age groups of healthcare professionals in the older age groups, a significant predominance of the proportion of seronegative subjects was observed (the parameter varied from 34.6% (95% CI: 27.1–42.7%) in the age group of 30–39 years to 50.5% (95% CI: 40.2–60.7%) in age group 50–59 years). This makes healthcare professionals potential sources of infection for children under one year of age.

A high proportion of pregnant women with IgG antibodies to diphtheria and tetanus, with a lower proportion of those with IgG antibodies to pertussis, was identified by researchers in Japan and Germany (32, 33). Thus, according to researchers, out of 100 pregnant women examined in Japan, 100% were sero-prevalent for diphtheria and tetanus and about 70% for pertussis. In Germany, out of 290 women, the proportion of persons with immunity to diphtheria and tetanus was 70% and 93%, respectively, while IgG antibodies to pertussis were found in 37% of the examined.

In our preliminary studies, a significant difference between the proportion of seronegative subjects and the timing of gestation in pregnant women was not established (38.6% (95% CI: 29.6–48.1%) in the first trimester, 31.1% (95% CI: 18.1–46.6%) in the second trimester, 31.7% (95% CI: 18.0–48.0%) in the third trimester). It is likely that the insignificant decrease in the levels of IgG antibodies by the third trimester is associated with the active transfer of antibodies to the fetus and the physiological increase in the volume of circulating blood in the mother. One of the debatable issues regarding the formation of sustainable immunity in newborns to whooping cough is the question of the period of primary immunization. The two most studied immunization tactics are (1) indirect immunization of infants through placental IgG antibodies when vaccinating pregnant women in the third trimester and (3) direct immunization in the first hours of a newborn's life (at birth). In relation to the first tactic, such aspects of vaccination as the optimal time of vaccine administration, its safety and interference of maternal antibodies remain under discussion (34). It is immunization in the first months of the third trimester (27–36 weeks of gestation) that is designated by researchers as the optimal time for Tdap vaccination, with the aim of subsequent transfer of maternal antibodies to the newborn (14). In relation to the second tactic, in general, little data has been collected on the effectiveness and safety of the proposed method, and the question of the type of vaccine administered (whole-cell vaccines or acellular vaccines) remains unresolved. A 2021 meta-analysis combining data from 29 studies showed that both immunization tactics are equally safe for both mother and child. It has been described that the use of whole-cell vaccines in neonates can lead to a reduction in induced immune paralysis and significantly reduce the levels of pertussis antibodies in infants before and after routine vaccination. However, it has been shown that the use of acellular vaccines does not lead to a decrease in antibodies prior to routine vaccination (35). Which immunization tactic is more effective and safer requires further study (8).

IgA antibodies to pertussis microbes were found in 12.5% of pregnant women. This finding allows us to suppose that in some of the subjects, immunity to pertussis is formed only within 12 months from the moment of contact with the pathogen and not as a result of the preservation of post-vaccination immunity, since in Russia there are no regulated booster vaccinations against pertussis in people older than 7 years. The proportion of high IgG is due to both the preservation of post-vaccination immunity and natural immunization as a result of pertussis. It must also be taken into account that immunity wanes considerably within 6 years of receiving a whole cell pertussis containing vaccine, so that a person would not have protective antibody titers after this time if he did not revaccinate. In this regard, we tried to find an association between the level of post-infection anti-pertussis IgA (along with post-vaccination anti-tetanus IgG and anti-diphtheria IgG) and the presence of a protective level of immunity to pertussis. To solve this problem, we built a predictive model using discriminant analysis using the stepwise selection method.

The conducted discriminant analysis, with the construction of the model (YIgG = 0.493 + 0.051 × XIgA − 0.056 × Xw; overall diagnostic significance was 70.2%) suggests that with the registration of high IgA values (taking into account the weeks of gestation), high values of IgG antibodies will also be recorded (Figure 1A). It is known that test systems have already been developed that can assess the level of IgA to pertussis in saliva (36). As a result, the proposed model can serve as the foundation for future larger population-based studies aimed at developing and implementing non-invasive methods for assessing pregnant women's immunological protection against pertussis. Such a non-invasive method for determining a protective level of immunity to pertussis would be in demand in those countries where pertussis booster vaccination national programs are not carried out, since low levels of specific IgG in pregnant women can predispose disease in the expectant mother and also be the reason for the lack of innate immunity in children under 2–3 months of age (Figure 1B).

Despite the absence of a high registration of pertussis among healthcare professionals, this professional group is at risk of being a source of infection for children under the age of one year (12). In this regard, the significant predominance of the proportion of diphtheria and pertussis seronegatives in healthcare professionals, when compared with pregnant women, is alarming. This may contribute to the formation of a nosocomial cluster of sick healthcare professionals, which, in turn, increases the risk of encountering a source of infection in children under one year of age. The findings are consistent with previously published findings that revealed relatively high proportions of seronegatives among medical personnel (36–38).

Considering the data obtained from the preliminary study, we believe that it is necessary to make certain changes to the national immunization programme in the Russian Federation. For example, it would be advisable to start vaccinating children not at three, but at two months of age, using a cell-free vaccine (Tdap), which is successfully practiced in several European countries (s.a. Germany, Spain, and France). The US CDC also recommends routine DTaP at 2, 4, and 6 months, at 15 through 18 months, and at 4 through 6 years (39).

Also, the rational solution would be to mandately revaccinate healthcare profession als working with children every ten years with the acellular pertussis vaccine, with legislative consolidation of this in the Russian Federation's current national immunization calendar for public health reasons.

## Conclusion

In the Moscow region (Solnechnogorsk city) of the Russian Federation, a preliminary cross-sectional study was conducted, which found that among pregnant women, the vast majority have a “high” level of IgG antibodies to diphtheria and tetanus, but only a third have a protective level of IgG antibodies to pertussis. A similar situation was found for healthcare professionals. However, the proportion of unprotected medical workers compared to pregnant women is lower. Because these two social groups are the primary source of diphtheria, pertussis, and tetanus infection in children under the age of one year, changes to the Russian Federation's current national immunization calendar are required.

## Data Availability

The original contributions presented in the study are included in the article/Supplementary Material, further inquiries can be directed to the corresponding authors.
